# Instances of Fish-Poison

**Published:** 1811-05

**Authors:** D. Quarrier

**Affiliations:** Surgeon. Princess Charlotte. H. M. Ship Princess Charlotte, Hamoaze


### *To the Editors of the Medical and Physical Journal.*

Gentlemen,

On the 15th September, 1809, whilst on a voyage to St. Helena, in lat. 00° 16′ S. long. 5° 47′ W. a large albicore was caught, and as we had been in the habit of eating the large rapacious fish of the northern hemisphere with impunity, nobody had the smallest suspicion of danger, more especially when so far from land, and it was eaten the first day without evincing any poisonons effect. On the 16th several of the midshipmen dined on it, I visited them about half an hour after, and found Mr. Crowe, a young gentleman of a full plethoric habit, ætat. 17, complaining of severe pain and giddiness in the head, drowsiness, violent pain and uneasiness at the pit of the stomach; great heat; much thirst; itchiness and difficulty in voiding his urine; his head was much swelled; his eyes were prominent and greatly inflamed; his face was much flushed; the belly was tumid, and a florid eruption extended itself over the neck, breast, belly, and extremities; the tongue was swelled and tremulous, and a burning sensation was felt all over it; the pulse was full, strong, and greatly accelerated.

An emetic was immediately given, together with plenty of diluents; a vast quantity of green bilious matter was vomited along with fish and other contents of the stomach, which was attended with immediate relief; a large dose of ol. ricin. was administered after the operation of the emetic, and an anodyne was taken in the evening; he was convalesced next morning and returned to duty in a few days. Several of the other gentlemen were affected in a similar manner, but none so severely; the same mode of treatment was adopted with success.

During our stay at St. Helena I had an opportunity of witnessing the same effects from eating the fish at that Island, more particularly the mackarel, which were caught in great abundance; almost all the officers, and a number of the seamen were poisoned by them; but it is a singular fact, that they were in no instance poisonous when eaten on the day they were taken, and were invariably so if kept during the night! the symptoms were the same as Mr. Crowe's, modified in proportion to the quantity used. I in no instance perceived the pungent heat and contraction of the æsophagus, which Dr. Chisholm mentions as distinguishing symptoms of the disease (see his ingenious remarks, Edinburgh Medical and Surgical Journal, vol. 4.) and which I have observed in the West Indies, but the heat of the mouth and tongue was excessive.

Copper is generally supposed to be the basis of fish poison; there were no banks near the place where the albicore was taken, and the nearest land (St. Matheo) was distant about one hundred and thirty miles; I should imagine, that if copper was so minutely diffused throughout the fish as to produce such violent effects, the exposure to the night air would not have added to its virulence; the same objection might be made to its dependance on the stomach or gall bladder. Dr. Chisholm observes, that "a gentleman of Antigua used to escape with impunity, by extracting the gall bladder without diffusing its contents over the abdomen." (Edin. Medical and Surgical Journal, vol. 4.) A number of smaller fishes were found in the stomach of the albicore, some of them half digested; it was cut up in junks shortly after being taken, therefore it is not likely or probable that the contents of the stomach or gall bladder could have been diffused over so large a fish*, and all parts were alike poisonous; even the mode of cooking made no alteration in its effects; but supposing the contents of either had been diffused over most parts of the fish, would its acrimony have been so much increased by one nights exposure?

The fishermen at St. Helena impute the poisonous quality of the fish to the influence of the moon, but I should suppose this opinion must have originated from their taking a greater number by moonlight than at any other time, and consequently are more likely to be kept longer than when less plentiful; the same opinion prevails amongst the fisherman at Bermuda.

I am inclined to believe that there is a certain poisonous principle inherent in all kinds of fish, and that its acrimony is increased more or less by the qualities of the substance upon which fish feed; by exposure to the air; and by disease and other casualties; and I find this opinion more firmly impressed on my mind, when I consider that even on our own shores, weak and irritable habits are often greatly disordered by eating what are commonly esteemed the most wholesome fish; however, I shall leave it to those particularly versed in physiological disquisition, to determine whether this deleterious property is dependant on the loss of oxygen, the absorption of azote, or upon the presence or absence of some other principle.

D. QUARRIER, Surgeon.
Princess Charlotte.

*H. M. Ship Princess Charlotte,*
*Hamoaze, Oct.* 25, 1810.

---

* It was five feet eight inches long, and three feet ten inches in circumference, and weighed one hundred and seventy-six pounds.

